# Use of over-the-counter mouthwashes as an additional measure in individual oral prophylaxis on adults with plaque-induced gingivitis: a double-blind, parallel, randomized controlled trial

**DOI:** 10.1186/s12903-023-03779-1

**Published:** 2024-01-16

**Authors:** Flavia Vitiello, Riccardo Monterubbianesi, Scilla Sparabombe, Denis Bourgeois, Vincenzo Tosco, Fahad Ali Alshehri, Florence Carrouel, Angelo Putignano, Giovanna Orsini

**Affiliations:** 1 Department of Clinical Sciences and Stomatology (DISCO), Università Politecnica delle Marche, Ancona, 60126 Italy; 2 Research Unit UR 4129, University Claude Bernard Lyon 1, University of Lyon, Lyon, 69008 France; 3 Department of Periodontics and Community Dentistry, College of Dentistry, King Saud University, Riyadh, 12372 Saudi Arabia; 4 National Institute of Health and Science of Aging (INRCA), Ancona, 60124 Italy

**Keywords:** Gingival bleeding, Biofilm, Bioflavonoids, Chlorhexidine, Inflammation, Mouthwash, Periodontology

## Abstract

**Background:**

Plaque-induced gingivitis is a chronic inflammatory condition characterized by complete reversibility of tissue damage once the periodontal biofilm has been disorganised. The aim of this study was to evaluate the efficacy of two commercially available mouthwashes (MWs) versus a chlorhexidine (CHX) 0.12% MW in reducing gingival bleeding (GB) in adults with plaque-induced gingivitis.

**Methods:**

The present study was a double-blind, parallel, randomized controlled trial involving 6492 gingival sites (i.e. 39 subjects × 28 teeth × 6 sites/tooth) aged 18–75 years. During a 2-week period, subjects were randomized to receive MWs: a control CHX 0.12% MW (group C, 1818 sites); a MW test containing CHX 0.09% + Citrox®/P complex (group CX, 2628 sites); a MW test based on natural compounds (group P, 2016 sites). GB was assessed at the inclusion visit (T_0_) and after 2 weeks of MW use (T_1_). Analyses of GB were compared between groups and then restricted to subjects with bleeding sites between 10 and 30% (moderate gingivitis) or ≥ 30% (severe gingivitis) at T_0_. Pairwise comparisons were made between groups and logistic regression was used to identify correlates of GB (T_1_).

**Results:**

For total bleeding site analysis, GB reduction between T_0_ and T_1_ ranged from 23% (C), 26% (CX) and 36% (P), respectively (all *p* < 0.05). Multiple comparison between groups showed that group C was significantly less effective (p < 0.05) than groups CX and P. Splitting the analysis, in patients with severe gingivitis (≥ 30% bleeding sites at T_0_), all MWs had a positive effect on GB with a reduction at T_1_ of 36% (C), 33% (CX) and 42% (P), respectively. While GB reduction between T_0_ and T_1_, was significant for all groups, the comparison among groups showed no significant difference between group C and CX, whereas the improvement was significant for group P. On the other hand, in adults with moderate gingivitis (< 30% bleeding sites at T_0_), only CX and P had a positive effect on GB reduction at T_1_(9% in CX and 2% in P, respectively), although the differences between the three groups were not significant.

**Conclusion:**

The daily use of MWs with natural components (groups P and CX) for 2 weeks should be considered positively as an adjunct to individual oral prophylaxis to reduce GB compared to the control MW containing CHX 0.12% (group C) in healthy adults with plaque-induced gingivitis. For subjects with severe gingivitis, it is advisable to first use natural MW (P) and then MW based on CHX 0.09% with natural components (CX), compared to MW with CHX 0.12% (C). For adults with moderate gingivitis, P and CX can be advisable, even if no definitive recommendations can be drawn.

**Trial Registration:**

ACTRN12622000215729, 07/02/2022.

## Introduction

Current strategies for fighting bacteria biofilms have been categorized into three principal approaches: (i) external forces application to eradicate the biofilm; (ii) modification of the properties of the sensitive surfaces to inhibit biofilm formation; and (iii) signal pathways regulation to induce inhibition of biofilm formation [1].

Plaque-induced gingivitis is an inflammatory response of the gingival tissues resulting from bacterial plaque accumulation, estimated at 1011 cells/mL, located at and below the gingival margin [2]. Gingival bleeding (GB) is a leading symptom of plaque-induced gingivitis [3]. If adequate and effective individual mechanical prophylaxis is applied, biofilm on accessible tooth surfaces will be disorganized. However, mechanical methods may be insufficiently feasible and/or suitable in case of patients with physical disabilities, who have difficulties in managing motor skills and find home dental care complicated [4–7]. Chemical preparations like antimicrobial mouthwashes (MWs) have therefore been suggested as a complement to, or replacement for mechanical plaque control [8]. In addition, due to their simple use and over-the-counter availability, MWs are also particularly well appreciated by consumers.

In oral health, chlorhexidine (CHX)-based MWs, broad-spectrum antiseptics with an effective remanence, are used prophylactically as well as therapeutically. For many years, CHX was considered as the main active ingredient providing effective, durable antibacterial action [9].

In the case of gingivitis and before any surgical procedures, recommendation of an over-the-counter antimicrobial MW with CHX 0.12%, as an adjunct to the mechanical disorganization of dental biofilm as part of an oral hygiene process is indicated [10]. However, CHX has several disadvantages including their tendency to stain teeth and leading to irritation of soft tissues as being the most common complaints [11]. Noteworthy, CHX had significant ecological impacts on the microbial contents of biofilm after a 7-day, twice-daily exposure [12]. To achieve a stronger impact, create a synergy and obtain a longer action of the substantivity, active components, antiseptics mostly of natural origin have been combined with CHX MWs. Natural molecules and nanoparticles having significant effects on dysbiotic biofilms in oral healthcare were also assessed [13, 14]. To overcome these, naturally occurring antimicrobial agents are being used individually or in combination [15, 16]. Research has recently focused on exploiting the positive properties of natural substances and mediators of periodontal inflammation as cost-effective, safe therapeutics [17]. Nowadays, the anti-inflammatory, antioxidant, antinociceptive effects and antimicrobial properties of these active gradients have been well documented [18].

Applied to plaque-induced gingivitis, there is insufficient evidence of an association between efficacy and the concentration level of CHX MW. Similarly, there is no consensus on the efficacy of CHX in reducing gingivitis in people with moderate or severe gingival inflammation risk [4]. Therefore, more homogenous RCTs with large sample size are needed to define the role of CHX [19].

The objective of this double The aim of this double-blind, parallel, randomized controlled trial was to clinically evaluate the efficacy of three MWs in reducing GB in adults with plaque-induced gingivitis: (i) a MW containing CHX 0.12% alone; (ii) a MW containing CHX 0.09%, hyaluronic acid (HA), polylysine and Citrox®; and (iii) a MW containing only natural extracts. The null hypothesis was that there would be no difference in the efficacy of reducing plaque-induced gingivitis between the three types of mouthwash evaluated. It is assumed that all interventions have a similar effect on the reduction of plaque-induced gingivitis.

## Materials and methods

### Study design

The study was designed as a double blind, parallel, randomized controlled trial. The guidelines of the CONSORT Statement were followed. The study was approved by the Ethical Committee of the Università Politecnica delle Marche (Ancona, Italy) (no. MTHW-CLX/HERB-2021) and registered in the New Zealand Clinical Trials Registry (trial number: ACTRN12622000215729, 07/02/2022). Written informed consent in accordance with the Declaration of Helsinki was obtained from all enrolled subjects.

### Participants

The population consisted of healthy volunteers aged 18–75 years. All candidates were screened for eligibility by the research team according to the inclusion and exclusion criteria.

The inclusion criteria were: (i) 18–75 years of age, (ii) gingivitis (> 10% sites with bleeding on probing), (iii) acceptance of the terms and conditions of the study, (iv) signing of the informed consent form.

Exclusion criteria were: (i) the presence of medical pathologies such as diabetes, haemophilia, anticoagulant treatment and risk of infectious endocarditis, (ii) immune deficiencies, (iii) any systemic disease affecting salivary flow, (iv) the presence of periodontal disease - stages I, II, III, IV, (v) the presence of active caries lesions; (v) presence of active caries lesions; (vi) fewer than 20 natural teeth; (vii) use of oral antiseptics in the previous 3 months; (viii) use of antibiotics or antimicrobial drugs within 3 months prior to enrolment; (ix) allergy to any of the components of the MWs tested; (x) presence of orthodontic appliances or dentures; (xi) history of allergy to any of the ingredients used in the study; (xii) pregnancy. Each subject was verbally informed about the products, the purpose and the protocol of the study. All subjects were allowed to withdraw from the study at any time.

### Interventions

The study was conducted in the Outpatient Department of Clinical Sciences and Stomatology (DISCO) of Università Politecnica delle Marche (Ancona, Italy) from 28th February 2022 to 30th April 2022. At baseline, the prescreened participants were referred to the dental clinic for baseline oral examinations. During an initial oral examination, a dentist verified the individual eligibility criteria (V.T.).

The participants were randomly assigned to one of the following three groups:

#### Group C

control, MW with CHX 0.12%;

#### Group CX

test, MW with CHX 0.09%, HA, polylysine and Citrox®/P complex, (PerioPlus+ Regenerate, Curaden, Kriens, Switzerland);

#### Group P

test, MW with natural extracts (Pural, Fitomedical snc, Binasco, Italy).

The detailed composition of the MWs used in this study is reported in Table 1.

**Table 1:** Description of the materials used in the study

Group		Ingredients
C	Control	Chlorhexidine Digluconate 0.12%
CX	Test	Aqua, Xylitol, Polysorbate 20, Chlorhexidine Digluconate 0.09%, Aroma, Phenoxyethanol, Vp/Va Copolymer, Sucralose, Cetylpyridinium Chloride, Polylysine, Citric Acid, Citrus Aurantium Amara Fruit Extract*, Glycerin, Sodium Hydroxide, Sodium Chloride
P	Test	*Propolis resin extract* (1:3), *Plantago lanceolata leaves extract* (1:10), 1.75% of essential oils from *Salvia officinalis*, *Salvia officinalis leaves extract* (1:1), *Mentha piperita leaves, Syzygium aromaticum buds, Pistacia lentiscus oleoresin* and *Commiphora myrrha oleoresin*.

All MWs were made indistinguishable by the absence of a label in 200 mL plastic bottles marked only with the patient’s number, so that both the patient and the experimenter were unaware of the type of MW. All participants received two bottles of their assigned products. Patients were instructed on how to use the MWs in combination with their home oral care habits according to their assigned group: Group C and CX, 10 mL undiluted twice daily (morning/evening) for 60 s after tooth brushing; Group P, 3–5 mL MW diluted with water according to the manufacturer’s instructions twice daily (morning/evening) for 60 s after tooth brushing [20]. A test rinse. A test rinse was performed during the study visits under expert supervision in the outpatient clinic. Subsequent rinsing with water was not allowed. Participants were advised to stop oral hygiene at least 8 h and not more than 18 h before the clinical examinations.

At the start of the study, each participant was given a tube of toothpaste with no effect on gingival inflammation. The composition of this SLS-free toothpaste used in the study included Amine fluoride; hydrated silica, aqua, sorbitol, hydroxyethylcellulose, polyethylene, titanium dioxide, olaflur, saccharin flavour, limonene (Elmex, Colgate-Palmolive Company, NY, USA). At the end of the study, patients were asked to return all bottles of MW.

### Outcome measures

The primary outcome was to evaluate the change in bleeding scores in adults with plaque-induced gingivitis after the use of three MWs from baseline (T_0_) to 14 days (T_1_).

The secondary outcomes were to evaluate the change in partial bleeding scores of the buccal and lingual/palatal sites, maxillary and mandibular teeth, anterior and posterior teeth, and interproximal sites in adults with plaque-induced gingivitis after the use of three MWs from baseline (T_0_) to 14 days (T_1_).

### Assessment

All selected subjects were followed for 2 weeks after the inclusion visit (T_0_). GB was assessed using a periodontal probe (PCP UNC 15, Hu-Friedy, Chicago, USA) [21] with firm and continuous pressure until maximum pressure was achieved with minimal discomfort to the subject. Probe placement pressure was approximately 50–100 N/cm^2^ (0.20–0.40 gram-force). After 30 s, each gingival unit was scored for bleeding: 0, no bleeding; and 1, bleeding. Probing started in the upper right quadrant and ended in the lower right quadrant. Bleeding scores were:

- *Total bleeding score*: the presence/absence of GB has been recorded at six sites per tooth (three on buccal tooth face and three on palatal/lingual tooth face). The results have been reported as percentage of the positive sites on the total surfaces [22]. Based on the percentage of total bleeding score at T_0_, we defined patients at high (> 30% bleeding sites) and low (< 30% bleeding sites) risk of gingivitis [23].
- *Partial bleeding score*: the values of total bleeding score were subdivided in partial bleeding scores of the buccal and lingual/palatal sites, of the maxillary and mandibular teeth, of the anterior and posterior teeth and of the interproximal sites [24].

A single trained and calibrated dental examiner (F.V.) performed all examinations.

### Randomization

A stratified (two levels for baseline bleeding and two levels for gender) block randomization method was used to ensure that the randomization process produced balanced groups with respect to the main covariates (gender and baseline bleeding). Stratified randomization was performed by creating a separate block for each covariate combination, and participants were allocated to the appropriate covariate block. Once all subjects had been identified and allocated to blocks, simple randomization was performed within each block to assign subjects to one of the groups. The participant was identified by a code.

There was a clear distinction between the generator of the allocation and the people responsible for carrying out the allocations. The implementation of the intervention was carried out by staff who were not involved in the data collection process. Participants and providers had no information about the ingredients of the MWs. To ensure impartiality, during the evaluation and analysis phase, statisticians, clinical research associates and clinicians were unaware of the group to which the participant belonged. Identification codes were securely held by the study manager and remained sealed until the end of the study to maintain confidentiality and avoid bias.

### Sample size calculation

Sample size was calculated using Sample Power 2.0 (SPSS, Chicago, IL, USA). The reduction in GB of the sites was considered as the main outcome variable. The sample size was based on the study of by Saliasi et al. [25], which reported a mean difference in GB between time points of 30% with a standard deviation of 5%.

Using this estimate, with an alpha risk of 5% for a Cohen’s d estimated at 0.5 and a statistical power of 80%, we obtain a minimum sample of 4990 sites, i.e. approximately 10 participants per group. In addition, considering that approximately 90% of the sites would be eligible for analysis, i.e. 10% would be excluded due to the presence of malocclusion or missing teeth, the sample size increases to 5544 sites.

### Statistical analysis

Demographic and baseline characteristics were compared between treatment groups using analysis of variance (ANOVA) or Chi-squared test. SPSS Windows 20.0 (IBM, Chicago, IL, USA) was used for descriptive statistics (percentages and means with SD) and analytical statistics (p-value calculation) in those analyses where the subject was the unit of analysis. XLSTAT 2022 (Addinsoft, Paris, France) was used for inferential statistics (p-value calculation) in analyses where the site was the unit of analysis to adjust for clustering (multiple sites within patients). The outcome variable was site of bleeding after use of MWs at the site level.

The statistical methods reported in Table 2 included: analysis of bleeding outcomes after treatment by both site and location. Standard errors (SEs) were corrected for complex sampling (multiple sites within the mouth) using the DESCRIPT procedure in XLSTAT 2022. P values were corrected for complex sampling (multiple sites within the mouth) using chi-squared analysis (CROSSTAB procedure in XLSTAT 2022). The percentage difference between control (C) and test (T) subjects was {[(%C - %T)/%C] x 100}. 95% CI was = % ± 1.96 SE, where the standard error (SE) was calculated after correction for multiple sites in the mouth (using the DESCRIPT procedure in XLSTAT 2022). When the global P-value was significant, paired comparisons were performed using chi-squared analysis, adjusted for complex sampling. All statistical tests were 2-sided with significance level alpha = 0.05.

**Table 2:** Distribution of individual characteristics at baseline (T_0_) (n = 39). C: Group control, CHX 0.12% MW; CX: Group test, CHX 0.09% + HA, polylysine, xylitol, and Citrox®; P: Group test, MW containing natural extracts

Variables	C	CX	P	Global *p*-value
Male, N(%)	5 (45.0%)	6 (37.5%)	3 (25.0%)	0.59
Female, N(%)	6 (55.0%)	10 (62.5%)	9 (75.0%)	0.59
Age (mean ± sd)	34.7 ± 13.2	31.6 ± 11.6	29.7 ± 7.9	0.06
Smokers, *N* (%)	2 (27.3%)	6 (55.0%)	2 (27.3%)	0.08
% Bleeding (mean ± sd)	39.31 ± 1.15	36.87 ± 0.94	39.04 ± 1.09	0.65
≥ 30% Bleeding sites	63.6%	56.2%	75.0%	0.22
< 30% Bleeding sites	36.4%	43.8%	25.0%	0.50

## Results

**Fig. 1:**
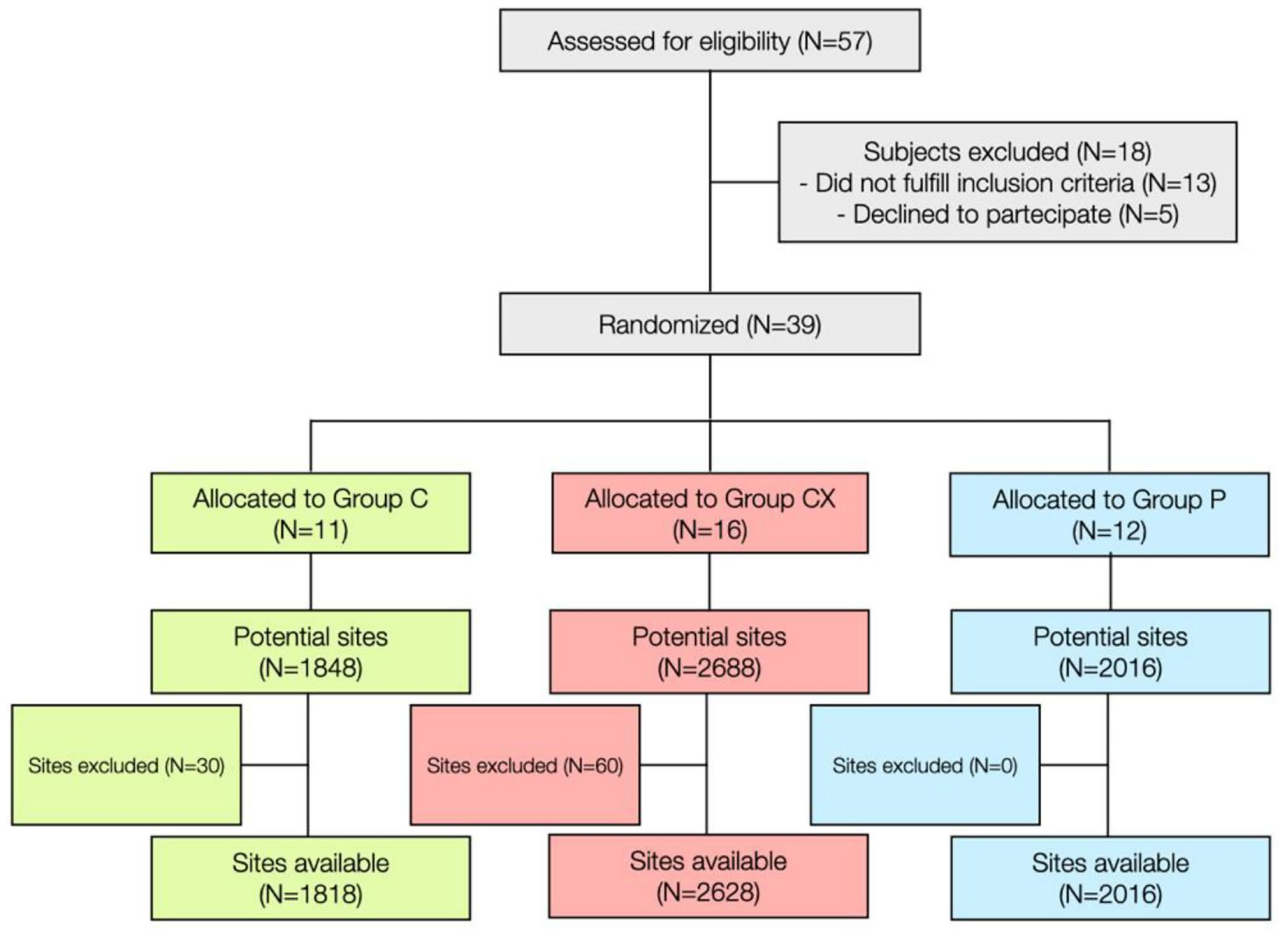
CONSORT flow diagram of the study

The CONSORT diagram for this study is shown in Fig. 1. Of the 57 adults, 13 were ineligible and 5 participants withdrew for reasons unrelated to the study protocol. This left 39 participants in the study. Of the 6552 a priori available sites (i.e. 39 subjects × 28 teeth × 6 sites/tooth), 90 sites were excluded due to missing teeth, leaving a total of 6462 sites.

All clinical parameters were normally distributed. The patients, 36% of whom were male with a mean age of 33.6 (± 12.6) years, had the characteristics described in Table 3. There were no significant differences in mean age, sex, or smoking between the two groups. No adverse events were observed in any patient during the study period.

**Table 3:** Bleeding scores according to the different sites and treatment group. C: Group control, CHX 0.12% MW; CX: Group test, CHX 0.09% + HA, polylysine, xylitol, and Citrox®; P: Group test, MW containing natural extracts

Time	C		CX		*P*		Paired Comparisons (*p*-Value)		
	*n*	% ± se	*n*	% ± se	*n*	% ± se	C-CX	C-P	CX-P
**Total bleeding sites**									
**T**_**0**_ **(Baseline)**	1818	39.31 ± 1.15	2628	36.87 ± 0.94	2016	39.04 ± 1.09	0.10	0.86	0.13
**T**_**1**_ **(2 weeks)**		30.13 ± 1.08		27.09 ± 0.87		24.9 ± 0.96	0.03	0.00	0.09
***p-value***		0.00		0.00		0.00			
**Effect. T**_**1**_, **% (95%-CI)**		23.36 ± 3.08		26.52 ± 2.51		36.21 ± 2.85			
**Anterior sites bleeding**									
**T**_**0**_ **(Baseline)**	594	23.91 ± 1.75	864	24.07 ± 1.46	648	26.08 ± 1.73	0.94	0.38	0.37
**T**_**1**_ **(2 weeks)**		18.69 ± 1.6		17.13 ± 1.28		14.66 ± 1.39	0.44	0.06	0.20
***p-value***		0.03		0.00		0.00			
**Effect. T**_**1**_, **% (95%-CI)**		21.83 ± 4.66		28.85 ± 3.80		43.79 ± 4.35			
**Posterior sites bleeding**									
**T**_**0**_ **(Baseline)**	1225	46.78 ± 1.43	1764	43.14 ± 1.18	1368	45.18 ± 1.35	0.06	0.41	0.25
**T**_**1**_ **(2 weeks)**		35.67 ± 1.37		31.97 ± 1.11		29.75 ± 1.24	0.03	0.00	0.18
***p-value***		0.00		0.00		0.00			
**Effect. T**_**1**_, **% (95%-CI)**		23.73 ± 3.88		25.89 ± 3.18		34.14 ± 3.58			
**Vestibular sites bleeding**									
**T**_**0**_ **(Baseline)**	909	36.74 ± 1.6	1314	34.47 ± 1.13	1008	38.59 ± 1.53	0.27	0.40	0.04
**T**_**1**_ **(2 weeks)**		32.12 ± 1.55		26.71 ± 1.22		20.34 ± 1.27	0.00	0.00	0.00
***p-value***		0.04		0.00		0.00			
**Effect. T**_**1**_, **% (95%-CI)**		12.57 ± 4.37		22.52 ± 3.51		47.3 ± 3.90			
**Lingual sites bleeding**									
**T**_**0**_ **(Baseline)**	910	41.87 ± 1.64	1314	39.27 ± 1.35	1008	39.48 ± 1.54	0.22	0.29	0.92
**T**_**1**_ **(2 weeks)**		28.13 ± 1.49		27.47 ± 1.23		29.46 ± 1.44	0.73	0.52	0.30
***p-value***		0.00		0.00		0.00			
**Effect. T**_**1**_, **% (95%-CI)**		32.81 ± 4.34		30.04 ± 3.58		25.38 ± 4.13			
**Maxillary sites bleeding**									
**T**_**0**_ **(Baseline)**	906	36.42 ± 1.6	1320	32.2 ± 1.29	1008	38.89 ± 1.54	0.04	0.27	0.00
**T**_**1**_ **(2 weeks)**		25.06 ± 1.44		20.53 ± 1.11		19.64 ± 1.25	0.01	0.00	0.60
***p-value***		0.00		0.00		0.00			
**Effect. T**_**1**_, **% (95%-CI)**		31.21 ± 4.22		36.24 ± 3.33		49.49 ± 3.89			
**Mandibular sites bleeding**									
**T**_**0**_ **(Baseline)**	913	42.17 ± 1.64	1308	41.59 ± 1.36	1008	39.19 ± 1.54	0.78	0.18	0.24
**T**_**1**_ **(2 weeks)**		35.16 ± 1.58		33.72 ± 1.31		30.16 ± 1.45	0.48	0.02	0.07
***p-value***		0.00		0.00		0.00			
**Effect. T**_**1**_, **% (95%-CI)**		16.62 ± 4.46		18.93 ± 3.70		23.04 ± 4.14			

*Effective size of the sample for each estimation. For instance, the first number value (*n* = 1818) is generated by the following calculation: 11 control subjects X 28 sites/patient X 6 sites/teeth = 1848 sites, minus sites with lack of tooth (*n* = 5), giving effective sample = 118 sites (= 1848 minus 30).

**Fig. 2:**
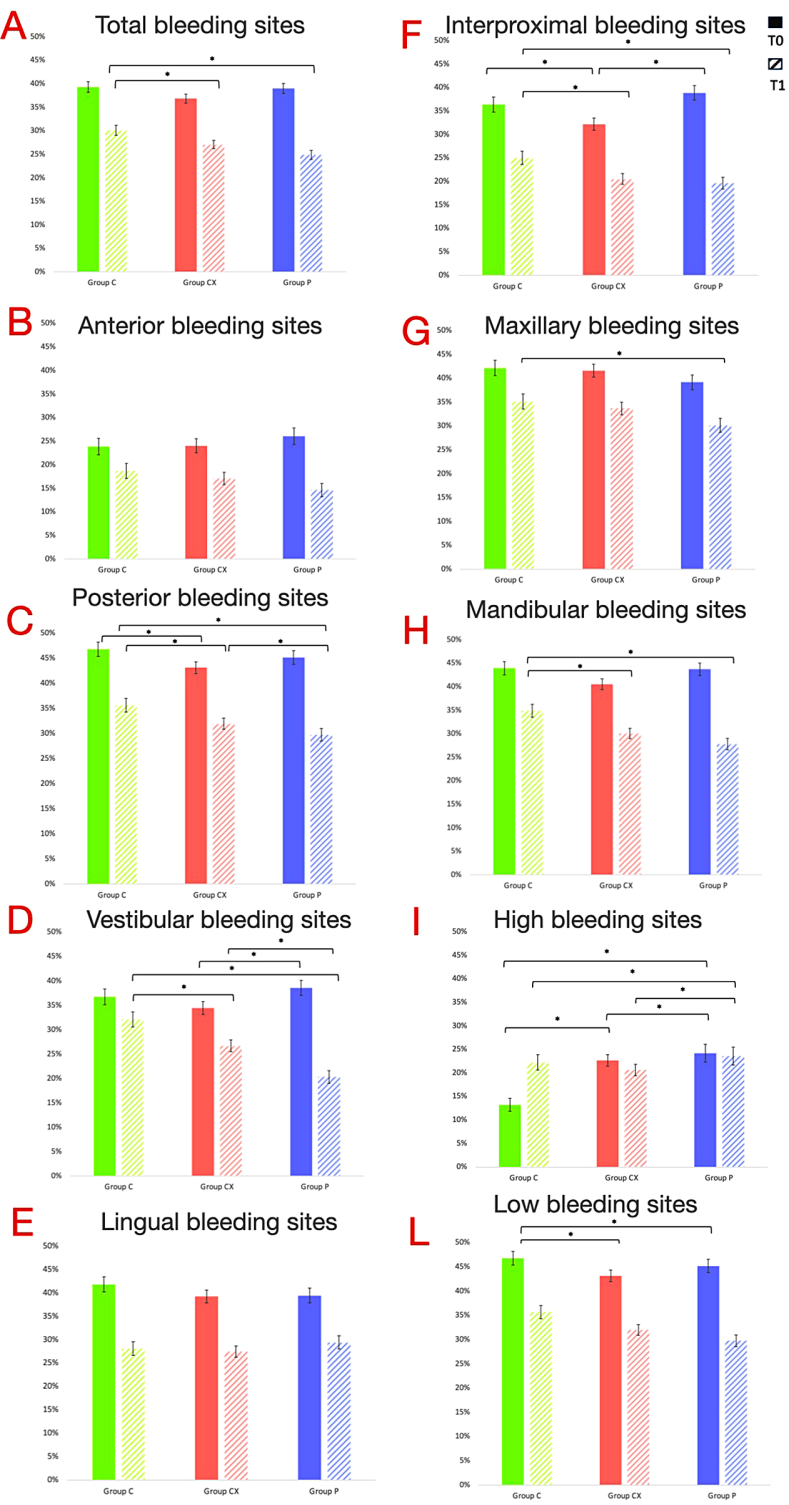
Graphical panel of changes in gingival bleeding in percentage (%) expressed as mean and standard deviation for each group from T_0_ to T_1_. Pairwise comparison test: the following level of statistical significance was considered: *p* < 0.05 (*). Comparisons between groups that are not statistically significant are not reported in the graph. Different colors were used for each group: Green, Group C (control, CHX 0.12% MW); red, Group CX (test, CHX 0.09% + HA, polylysine, xylitol, and CITROX®); blue, Group P (test, MW containing natural extracts) respectively. Different letters (**A-L**) were used to highlight the different data analyses

The analysis of bleeding scores after treatment is shown in Table 2; Fig. 2, both by site and by location. For all groups, the percentage of bleeding at T_0_ observed for each group was high (> 30%). The reduction in bleeding over the 2-week period in all sites was high for all MWs (23% (C), 26% (CX) and 36% (P)), with a 13% reduction observed between C and P and a 10% reduction between C and P. The paired comparison concluded that there were significant differences between C-CX and C-P, while CX and P had similar efficacy.

To better understand the results of the total bleeding scores, the indices were divided by blocks (anterior, posterior, vestibular and lingual) or arches (maxillary and mandibular).

The anterior site (incisor-canine block) bleeding scores between groups at T_0_ showed no significant differences in the percentage of bleeding, which can be considered moderate (< 30%). At T_1_, the between-group reduction in bleeding was not significant for C (22%, *p* = 0.03) and highly significant (*p* < 0.001) for CX and P (29% and 44%). The efficacy of C was lower than that of CX and P, but no statistically significant differences were found between the 3 groups.

Bleeding rates in the posterior sites (premolar-molar block) at T_0_ were high at around 45% in all groups. Despite a highly significant reduction in the percentage of bleeding between T_0_ and T_1_ for all groups (C, 24% vs. CX, 26% vs. P, 34%), group C is significantly less effective than the other two.

At the vestibular sites, all MWs had a significant effect on reducing bleeding at T_1_ (13%; 23%; 47%). We can conclude a significant superiority effect of the MW composed of natural ingredients (P) for the reduction of GB compared to group C (*p* < 0.05) and CX (*p* < 0.001). On the other hand, CX improved GB more than group C (*p* < 0.001).

On the other hand, while the reduction of GB at the lingual sites by approximately 35% between T_0_-T_1_ was significant for all groups (*p* = 0.00), they all had the same effect in terms of power (C = CX = P).

The effect of three MWs on improving GB in maxillary bleeding sites was highly significant (*p* = 0.00), with differences of 31%, 36% and 49%, respectively. The largest difference in GB reduction is observed between P and C (18%), while pairwise comparison analysis indicates that C = CX, C≠P and CX = P. For mandibular bleeding sites pairwise comparison analysis shows that C≠CX, C≠P and CX = P.

Table 4 presents the results obtained from the evaluation of the GB of the interproximal sites. GB decrease was evident in all groups between T_0_ and T_1_. Group C showed a statistically significant difference compared to the other groups CX and P. No difference was found between groups CX and P.

**Table 4:** Interproximal bleeding scores according to the treatment groups C: Group control, CHX 0.12% MW; CX: Group test, CHX 0.09% + HA, polylysine, xylitol, and CITROX®; P: Group test, MW containing natural extracts

Time	C		CX		*P*		Paired Comparisons (*p*-Value)		
	*n*	% ± se	*n*	% ± se	*n*	% ± se	C-CX	C-P	CX-P
**Interproximal sites bleeding**									
**T**_**0**_ **(Baseline)**	1224	43.95 ± 1.42	1768	40.55 ± 1.17	1356	43.73 ± 1.35	0.06	0.91	0.07
**T**_**1**_ **(2 weeks)**		34.89 ± 1.36		30.09 ± 1.09		27.8 ± 1.22	0.00	0.00	0.16
***p-value***		0.00		0.00		0.00			
**Effect. T**_**1**_, **% (95%-CI)**		20.63 ± 3.86		25.8 ± 3.13		36.42 ± 3.56			

Based on our results we divided the total bleeding scores as: a low level of bleeding if the participant had between 10 and 30% bleeding sites (moderate gingivitis) [26] and a high level of bleeding if the participant had ≥ 30% bleeding sites (severe gingivitis). In patients with ≥ 30% bleeding sites at T_0_, all MWs had a positive effect on GB reduction after 2-week period, with superiority observed for Group P (42%). Group P showed statistically significant differences from Group C and CX (Table 5). The groups with CHX as active ingredient (Group C and CX) had a comparable effect on GB (*p* = 0,23). In patients with < 30% bleeding sites at T_0_, in the group C a significant worsening of inflammation and gingival health was observed, with a 68% increase in the number of bleeding sites after 2 weeks of use. However, this result is not very reliable due to the difference at T_0_ between groups C (13%), CX (23%) and P (24%). Group CX and P were not effective in reducing bleeding rates (*p* > 0.05).

**Table 5:** Bleeding sites score according to patient’s baseline gingival bleeding and treatment groups. C: Group control, CHX 0.12% MW; CX: Group test, CHX 0.09% + HA, polylysine, xylitol, and Citrox®; P: Group test, MW containing natural extracts

Time	C		CX		P		Paired Comparisons (*p*-value)		
	*n*	% ± se ^b^	*n*	% ± se ^b^	*n*	% ± se ^b^	C-CX	C-P	CX-P
**Patient’s Bleeding at T**_**0**_ ** ≥ 30%**									
**T**_**0**_ **(Baseline)**	1176	53.57 ± 1.45	1464	48.16 ± 1.31	1512	43.98 ± 1.28	0.00	0.00	0.02
**T**_**1**_ **(2 weeks)**		34.44 ± 1.39		32.24 ± 1.22		25.33 ± 1.12	0.23	0.00	0.00
***p-value***		0.00		0.00		0.00			
**Effect. T**_**1**_, **% (95%-CI)**		35.71 ± 3.94		33.05 ± 3.51		42.41 ± 3.33			
**Patient’s Bleeding at T**_**0**_ **< 30%**									
**T**_**0**_ **(Baseline)**	643	13.22 ± 1.34	1164	22.68 ± 1.23	504	24.21 ± 1.91	0.00	0.00	0.50
**T**_**1**_ **(2 weeks)**		22.24 ± 1.64		20.62 ± 1.19		23.61 ± 1.89	0.42	0.58	0.17
***p-value***		0.00		0.23		0.82			
**Effect. T**_**1**_, **% (95%-CI)**		-68.24 ± 4.15		9.09 ± 3.35		2.46 ± 5.28			

Significant differences were observed between the different risk groups for the studied variables and sites bleeding at T_1_ (Table 6).

**Table 6:** Distribution of variables at total bleeding sites level and multivariate associations in the separate bleeding conditions (≥ 30% of Bleeding Sites at T_0_ and < 30% of Bleeding Sites at T_0_) groups between the variables studied and bleeding at T_1_ (2 weeks). C: Group control, CHX 0.12% MW; CX: Group test, CHX 0.09% + HA, polylysine, xylitol, and Citrox®; P: Group test, MW containing natural extracts. OR: odds ratio; CI: confidence interval

Variable	≥ 30% of Bleeding Sites at T_0_			< 30% of Bleeding Sites at T_0_		
	*n*	OR (95%-CI)	*p*-value	*n*	OR (95%-CI)	*p*-value
**Group C**	143	1.0		405	1.0	
**Group CX**	240	0.91 (0.03; 24.71)	0.05	472	0.91(0.09; 8.97)	0.03
**Group P**	119	1.08 (0.21; 5.66)	0.06	383	0.68(0.1; 4.16)	0.00

Based on our results, we divided the total bleeding score into: low bleeding if the participant had between 10% and 30% bleeding sites (moderate gingivitis) [24] and high bleeding if the participant had ≥ 30% bleeding sites (severe gingivitis). In patients with ≥ 30% bleeding sites at T_0_, all MWs had a positive effect on GB reduction at 2 weeks, with superiority observed for Group P (42%). Group P showed statistically significant differences from groups C and CX (Table 5). The groups with CHX as active ingredient (groups C and CX) had a comparable effect on GB (*p* = 0.23). In patients with < 30% bleeding sites at T_0_, a significant worsening of inflammation and gingival health was observed in group C, with a 68% increase in the number of bleeding sites after 2 weeks of use. However, this result is not very reliable due to the difference at T_0_ between groups C (13%), CX (23%) and P (24%). Groups CX and P were not effective in reducing bleeding rates (*p* > 0.05).

## Discussion

This original clinical trial was designed to assess the effectiveness of two different types of MWs (Groups CX and P) compared to 0.12% CHX one (Group C) on reducing GB after 2-week period of use in addition to the daily habits of home oral hygiene in adults with plaque-induced gingivitis. Our research was based on the 2018 classification standardized the identification of patients with intact periodontium who would be clinically diagnosed with gingivitis in terms of prevalence and severity (EFP/AAP, 2018 [26]). To investigate the prevalence and severity of gingivitis, GB was recorded on all teeth in order to define and classify gingivitis. Reduced GB means that gingival inflammation is under control, helping to prevent the progression of periodontal disease from reversible to irreversible. Bacterial plaque control is essential for the primary prevention of periodontitis [3, 27].

After 2 weeks of unsupervised use of MW, regardless of the category of MW, groups C, CX and P showed a significant reduction in GB, varying by 23%, 26% and 36% respectively. Firstly, we can conclude that these MWs help to reduce gingival inflammation and can be used as an adjunct to mechanical toothbrushing in the general population with plaque-induced gingivitis, i.e. >10% of bleeding sites. However, the CHX 0.12% rinse concentration in group C is significantly less effective than the other two products. This trend was found in almost all sub-analyses according to site location (maxillary, mandibular, vestibular, lingual).

Therefore, the proposed null hypothesis that the MW containing CHX 0.09%, hyaluronic acid (HA), polylysine and Citrox®, the MW containing natural extracts and the MW containing CHX 0.12% showed no differences in the reduction of GB in adults with plaque-induced gingivitis is rejected.

To further analyze the effect of the MWs on GB, the study population was divided into populations with 10–30% and ≥ 30% bleeding sites at baseline. In populations with < 30% bleeding sites, there were no statistical differences between the groups. Therefore, we can conclude that in adults with plaque-induced gingivitis with < 30% bleeding sites, the indication for MWs as used in the clinical trial does not add value.

Conversely, the most relevant indication concerns the recommendation of natural MW for subjects with ≥ 30% bleeding sites. In group P, a significant reduction of 42% was observed, showing significant superiority over the CHX groups (groups C and CX). This is the only result from the overall analysis and the site subanalyses performed in our study that highlights the clear superiority of natural MW over CHX 0.09% + natural components MW (group CX).

Our results do not allow us to explain which components of group P have the greatest effect on reducing GB compared to the other two groups containing CHX (groups C and CX).

Several hypotheses could be formulated to explain the results obtained. One could be related to the influence of the CHX concentration on the ability to form biofilms [4]. Another hypothesis would be to emphasise the added value of the association of CHX with natural components of the CX group (HA, polylysine, Citrox®/P complex and others), which would complement the biocidal action of CHX 0.09% by promoting the haemostasis of GB.

In particular, HA, contained in Group CX, is a natural biocompatible, biodegradable, non-toxic substance with multiple physiological functions, including tissue growth, bio-lubrication, modulation of water diffusion, maintenance of vascular elasticity, and promotion of wound healing after oral surgery [28, 29].

Flavonoids refer to a series of chemical compounds with two variable phenolic structure which show various bioactive functions including antioxidant, antiviral, antibacterial and anti-inflammation [30, 31]. Many flavonoids and tannins, as single compounds or in mixtures as natural extracts, are effective agents against bacteria responsible for periodontal disease, and other oral infections, considering their availability, efficacy, safety, and finally, the patient compliance.

So, the Citrox, an all-natural antiseptic agent, and polylysine, another naturally-occurring long-lasting antimicrobial, inhibit the proliferation of most microorganisms [32–35]. Polylysine is a non-specific cell attachment factor which facilitates cell adhesion on solid substrates by increasing electrostatic interactions of negatively charged ions on both the culture surface and cell membrane. Poly-L-lysine, when adsorbed on the culture surface, significantly increases the availability of positively charged sites for cell adhesion [32].

In this light, the scientific research aims to deepen the knowledge on the bacterial resistance option developed against CHX and experiment natural alternative products for the treatment and prevention of GB. In this regard, the introduction of herbal MW has gained popularity over chemical MWs due to their non-staining, non-irritant characteristics, and beneficial properties in periodontal inflammation as safe and cost-effective therapeutics [20, 31, 36]. They have minimal or no adverse effects. The discovery of natural compounds targeting the host immune responses offer promise to sustain health and improve clinical outcomes. Like dressings within a wound-healing process, the current trend in the creation of MW products has been to combine materials with outstanding benefits necessary for the wound-healing process, such as intrinsic antibacterial properties, high biological compatibility, environmental friendliness [37]. In addition, MWs are loaded with bioactive components, including growth factors, plant extracts, essential oils, antioxidants, anti-inflammatory agents and vitamins, to enhance therapeutic results, such as *Propolis resin extract*, *Plantago lanceolata*, *Salvia officinalis* leaves extract.

Several studies have reported the benefits of *Propolis* with regards to the reduction of the plaque index [38]. Giammarinaro et al., have studied the efficiency of Propolis versus CHX on a 40-patient sample with gingivitis, and found no significant differences between the test and the active group in bleeding on probing, probing pocket depth, and plaque index [39]. Nevertheless, patients who were treated with propolis had better results regarding oxidative stress markers in the saliva, with notable improvement of their periodontal health.

Anauate-Netto demonstrated that typified *Propolis* rinse was effective in reducing gingival inflammation with unsupervised rinsing twice a day for 28 days [40]. Furthermore, a meta-analysis revealed that the application of different components above-mentioned as CHX adjuvants improved the bleeding clinical parameters [41]. Accordingly, our positive results of both natural MW and MW combined with natural components could be due to their antioxidant action.

A major limitation of this study is the imbalance in the number of participants between groups. This imbalance may introduce a potential bias in the interpretation of the results. However, using the statistical unit based on the number of sites per person significantly increases the power of the test, which is close to 1.

This may be an advantage for detecting smaller effects or compensating for unequal sample sizes. Another limitation of the study is that no other periodontal indices were collected. On the other hand, it would have been useful to assess the reduction in GB midway through the study in order to redefine the duration of prescription and/or use. Similarly, the result of the “evolution of GB” test is a clinical criterion for evaluating the efficacy of MWs, but the assessment of the subjects’ perception of the MW assigned by randomization by means of a satisfaction questionnaire would have provided additional information for prescribing or recommending them, especially between the CX and P groups.

## Conclusion

The results of our study demonstrated the positive effect of the natural MW (group P) and the MW containing CHX 0.09% with natural components (group CX) on the reduction of GB, compared with the control MW containing CHX 0.12% (group C), in healthy adults with plaque-induced gingivitis.

In patients with severe gingivitis, it is recommended first to use natural MW (group P), then the MW with CHX 0.09% in combination with natural components (CX), compared to the control group (C).

On the other hand, in patients with moderate gingivitis, P and CX can be advisable, even if no definitive recommendations can be drawn. In addition to the use of MWs, biofilm disorganization on accessible dental surfaces and in interdental spaces is a necessary measure to control and reduce plaque-induced gingivitis. Further studies are needed to investigate the behaviour of MWs in balancing the oral microbiome, promoting symbiosis or causing dysbiosis, and to improve our understanding of the efficacy of both natural and non-natural components.

## Data Availability

The datasets used and/or analyzed during the current study are available from corresponding author on reasonable request.

## References

[1] Yin W, Xu S, Wang Y, Zhang Y, Chou SH, Galperin MY (2021). Ways to control harmful biofilms: prevention, inhibition, and eradication. Crit Rev Microbiol.

[2] Zayed N, Boon N, Bernaerts K, Chatzigiannidou I, Van Holm W, Verspecht T (2022). Differences in chlorhexidine mouthrinses formulations influence the quantitative and qualitative changes in in-vitro oral biofilms. J Periodontal Res.

[3] Murakami S, Mealey BL, Mariotti A, Chapple ILC (2018). Dental plaque-induced gingival conditions. J Periodontol.

[4] Hv PJ, C W. P, M H, T L, A C, Chlorhexidine mouthrinse as an adjunctive treatment for gingival health. The Cochrane database of systematic reviews [Internet]. 2017 Mar 31 [cited 2023 Jul 12];3(3). Available from: https://pubmed.ncbi.nlm.nih.gov/28362061/. 10.1002/14651858.CD008676.pub2 PMC6464488 28362061

[5] Silverman S, Wilder R (2006). Antimicrobial mouthrinse as part of a comprehensive oral care regimen. Safety and compliance factors. J Am Dent Assoc.

[6] Marruganti C, Gaeta C, Romandini M, Ferrari Cagidiaco E, Parrini S, Discepoli N et al. Multiplicative effect of stress and poor sleep quality on periodontitis: a university-based cross-sectional study. J Periodontol. 2023. 10.1002/JPER.23-0209 37477025

[7] Sparabombe S, Roncati M, Monterubbianesi R, Catellani A, Manzoli L, Bambini F (2018). Assessment of antiplaque effectiveness of chlorhexidine-soaked gauze compared to chlorhexidine mouth rinse: randomized clinical trial. J Investig Clin Dent.

[8] Jones CG (1997). Chlorhexidine: is it still the gold standard?. Periodontol 2000.

[9] Poppolo Deus F, Ouanounou A (2022). Chlorhexidine in Dentistry: Pharmacology, uses, and adverse effects. Int Dent J.

[10] Brookes ZLS, Belfield LA, Ashworth A, Casas-Agustench P, Raja M, Pollard AJ (2021). Effects of chlorhexidine mouthwash on the oral microbiome. J Dent.

[11] Mathew M, Joyshree C, Ratan VJ, Kartheek V, Thirumalai S, Banothu MN (2022). Anti-plaque efficacy of Hi-Ora mouthrinse and 0.12% chlorhexidine gluconate in patients with chronic gingivitis: a case–control study. J Oral Maxillofac Pathol.

[12] Bescos R, Ashworth A, Cutler C, Brookes ZL, Belfield L, Rodiles A (2020). Effects of Chlorhexidine mouthwash on the oral microbiome. Sci Rep.

[13] Carrouel F, Viennot S, Ottolenghi L, Gaillard C, Bourgeois D (2020). Nanoparticles as Anti-Microbial, anti-inflammatory, and remineralizing agents in oral care cosmetics: a review of the current Situation. Nanomaterials (Basel).

[14] Kanouté A, Dieng SN, Diop M, Dieng A, Sene AK, Diouf M (2022). Chemical vs. natural toothpaste: which formulas for which properties? A scoping review. J Public Health Afr.

[15] Al-Maweri SA, Nassani MZ, Alaizari N, Kalakonda B, Al-Shamiri HM, Alhajj MN (2020). Efficacy of aloe vera mouthwash versus chlorhexidine on plaque and gingivitis: a systematic review. Int J Dent Hyg.

[16] Chinsembu KC (2016). Plants and other natural products used in the management of oral Infections and improvement of oral health. Acta Trop.

[17] Lee CT, Teles R, Kantarci A, Chen T, McCafferty J, Starr JR (2016). Resolvin E1 reverses experimental Periodontitis and Dysbiosis. J Immunol.

[18] Murray MC, Worthington HV, Blinkhorn AS (1997). A study to investigate the effect of a propolis-containing mouthrinse on the inhibition of de novo plaque formation. J Clin Periodontol.

[19] Liu T, Chen YC, Jeng SL, Chang JJ, Wang JY, Lin CH (2023). Short-term effects of Chlorhexidine mouthwash and listerine on oral microbiome in hospitalized patients. Front Cell Infect Microbiol.

[20] Sparabombe S, Monterubbianesi R, Tosco V, Orilisi G, Hosein A, Ferrante L et al. Efficacy of an All-Natural Polyherbal Mouthwash in Patients With Periodontitis: A Single-Blind Randomized Controlled Trial. Front Physiol [Internet]. 2019 [cited 2019 Jun 4];10. Available from: https://www.frontiersin.org/articles/10.3389/fphys.2019.00632/full. 10.3389/fphys.2019.00632 PMC6540781 31191341

[21] Loe H, Silness J (1963). PERIODONTAL DISEASE IN PREGNANCY. I. PREVALENCE AND SEVERITY. Acta Odontol Scand.

[22] Bentley CD, Disney JA (1995). A comparison of partial and full mouth scoring of plaque and gingivitis in oral hygiene studies. J Clin Periodontol.

[23] Trombelli L, Farina R, Silva CO, Tatakis DN (2018). Plaque-induced gingivitis: case definition and diagnostic considerations. J Clin Periodontol.

[24] Bourgeois D, Saliasi I, Llodra JC, Bravo M, Viennot S, Carrouel F (2016). Efficacy of interdental calibrated brushes on bleeding reduction in adults: a 3-month randomized controlled clinical trial. Eur J Oral Sci.

[25] Saliasi I, Llodra JC, Bravo M, Tramini P, Dussart C, Viennot S (2018). Effect of a Toothpaste/Mouthwash Containing Carica papaya Leaf Extract on Interdental Gingival bleeding: a Randomized Controlled Trial. Int J Environ Res Public Health.

[26] Chapple ILC, Mealey BL, Van Dyke TE, Bartold PM, Dommisch H, Eickholz P (2018). Periodontal health and gingival Diseases and conditions on an intact and a reduced periodontium: Consensus report of workgroup 1 of the 2017 World workshop on the classification of Periodontal and Peri-implant Diseases and conditions. J Periodontol.

[27] Bourgeois D (2023). Next preventive strategies for oral health: evolution or revolution?. Front Public Health.

[28] Maria de Souza G, Elias GM, Pereira de Andrade PF, Andrade Sales KN, Galvão EL, Moreira Falci SG. The Effectiveness of Hyaluronic Acid in Controlling Pain, Edema, and Trismus After Extraction of Third Molars: Systematic Review and Meta-Analysis. J Oral Maxillofac Surg. 2020;78(12):2154.e1-2154.e12. 10.1016/j.joms.2020.07.005 32771444

[29] Birajdar MS, Joo H, Koh WG, Park H (2021). Natural bio-based monomers for biomedical applications: a review. Biomater Res.

[30] Chu M, Xu L, Zhang MB, Chu ZY, Wang YD (2015). Role of Baicalin in Anti-influenza Virus A as a potent inducer of IFN-Gamma. Biomed Res Int.

[31] Lee YJ, Beak SY, Choi I, Sung JS (2017). Quercetin and its metabolites protect hepatocytes against ethanol-induced oxidative stress by activation of Nrf2 and AP-1. Food Sci Biotechnol.

[32] Shi C, He Y, Feng X, Fu D (2015). ε-Polylysine and next-generation dendrigraft poly-L-lysine: chemistry, activity, and applications in biopharmaceuticals. J Biomater Sci Polym Ed.

[33] Carrouel F, Valette M, Gadea E, Esparcieux A, Illes G, Langlois ME (2021). Use of an antiviral mouthwash as a barrier measure in the SARS-CoV-2 transmission in adults with asymptomatic to mild COVID-19: a multicentre, randomized, double-blind controlled trial. Clin Microbiol Infect.

[34] Carrouel F, Conte MP, Fisher J, Gonçalves LS, Dussart C, Llodra JC (2020). COVID-19: a recommendation to examine the Effect of Mouthrinses with β-Cyclodextrin combined with Citrox in preventing Infection and progression. J Clin Med.

[35] Carrouel F, Gonçalves LS, Conte MP, Campus G, Fisher J, Fraticelli L (2021). Antiviral activity of reagents in Mouth rinses against SARS-CoV-2. J Dent Res.

[36] D’Ambrosio F, Di Spirito F, Amato A, Caggiano M, Lo Giudice R, Martina S (2022). Attitudes towards antibiotic prescription and Antimicrobial Resistance awareness among Italian dentists: what are the milestones? Healthcare. (Basel).

[37] Nguyen HM, Ngoc Le TT, Nguyen AT, Thien Le HN, Pham TT. Biomedical materials for wound dressing: recent advances and applications. RSC Adv 13(8):5509–28. 10.1039/d2ra07673j PMC9924226 36793301

[38] López-Valverde N, Pardal-Peláez B, López-Valverde A, Flores-Fraile J, Herrero-Hernández S, Macedo-de-Sousa B (2021). Effectiveness of Propolis in the treatment of Periodontal Disease: updated systematic review with Meta-analysis. Antioxid (Basel).

[39] Giammarinaro E, Marconcini S, Genovesi A, Poli G, Lorenzi C, Covani U (2018). Propolis as an adjuvant to non-surgical periodontal treatment: a clinical study with salivary anti-oxidant capacity assessment. Minerva Stomatol.

[40] ANAUATE-NETTO C, ANIDO-ANIDO A, LEEGOY HR, MATSUMOTO R, ALONSO RCB, MARCUCCI MC (2014). Randomized, double-blind, placebo-controlled clinical trial on the effects of propolis and chlorhexidine mouthrinses on gingivitis. Braz Dent Sci.

[41] Castro MML, Duarte NN, Nascimento PC, Magno MB, Fagundes NCF, Flores-Mir C (2019). Antioxidants as adjuvants in Periodontitis Treatment: a systematic review and Meta-analysis. Oxid Med Cell Longev.

